# Free school meals as an approach to reduce health inequalities among 10–12- year-old Norwegian children

**DOI:** 10.1186/s12889-019-7286-z

**Published:** 2019-07-16

**Authors:** Frøydis N. Vik, Wendy Van Lippevelde, Nina C. Øverby

**Affiliations:** 1 Department of Nutriton and Public Health, University of Agder, Post-box 422, N-4604 Kristiansand, Norway; 2 Department of Marketing, Innovation and Organisation, Ghent University, Ghent, Belgium

**Keywords:** Children, Free school meal, Intervention, Weight status, Healthy food score, Socio-economic status, Norway

## Abstract

**Background:**

Children spend a considerable amount of time at school and consume at least one meal/day. This study aimed to investigate if a free, healthy school meal every day for one school year was associated with children’s intake of healthy foods at school, weight status and moderating effects of socio-economic status.

**Methods:**

A non-randomized study design with an intervention and a control group was used to measure change in children’s dietary habits at lunchtime. In total, 164 children participated; 55 in the intervention group and 109 in the control group (baseline). Intervention-children were served a free, healthy school meal every school day for one year. Participating children completed a food frequency questionnaire at baseline, at five months follow-up and after one year. Children’s anthropometrics were measured at all three timepoints. Intervention effects on children’s Healthy food score, BMI z-scores, and waist circumference were examined by conducting a Repeated Measures Multivariate ANOVA. Moderating effects of children’s gender and parental socio-economic status were investigated for each outcome.

**Results:**

A significant intervention effect on children’s outcomes (multivariate) between baseline and after one year (*F* = 2.409, *p* < 0.001), and between follow-up 1 at five months and after one year (*F* = 8.209, *p* < 0.001) compared to the control group was found. The Univariate analyses showed a greater increase in the Healthy food score of the intervention group between baseline and follow-up 1 (*F* = 4.184, *p* = 0.043) and follow-up 2 (*F* = 10.941, *p* = 0.001) compared to the control group. The intervention-children had a significant increase in BMI z-scores between baseline and follow-up 2 (*F* = 10.007, *p* = 0,002) and between follow-up 1 and 2 (*F* = 22.245, *p* < 0.001) compared to a decrease in the control-children. The intervention-children with lower socio-economic status had a significantly higher increase in Healthy food score between baseline and follow-up 2 than the control-children with lower socio-economic status (difference of 2.8 versus 0.94), but not among children with higher socio-economic status.

**Conclusions:**

Serving a free school meal for one year increased children’s intake of healthy foods, especially among children with lower socio-economic status. This study may contribute to promoting healthy eating and suggests a way forward to reduce health inequalities among school children.

**Trial registration:**

ISRCTN61703361. Date of registration: December 3rd, 2018. Retrospectively registered.

## Background

A healthy diet is fundamental to health. A healthy diet among children and adolescents also protects against non-communicable diseases (NCDs) later in life [1]. Healthy dietary habits established early in life tend to persist into adulthood and thereby promote lifelong health [2, 3]. Childhood obesity is considered a serious public health challenge and tends to track into adolescence and adulthood [4–6]. A healthy diet is considered a main driver to sustain a healthy weight throughout life [3].

In a public health perspective, schools are ideal settings to promote healthy eating habits early in life since children consume at least one main meal per day at school. Socio-economic status (SES) in families is associated with children’s diet, i.e., lower SES families tend to have more unhealthy dietary habits [7, 8]. In Norway, the vast majority of children (96%) attend public schools [9]. Thus, socio-economic inequalities in healthy eating/health may be reduced if all children eat a free, healthy meal at school [10]. In Norway there is in general no school meal arrangement where food is provided (neither free nor parent paid), and the children typically bring packed lunch from home. Norwegian school children traditionally eat a cold bread meal during school hours [11]. Challenges with the traditional packed lunch are that some children bring an unhealthy lunch to school, and children may also skip lunch due to not bringing any lunch [12]. In 2015, renewed and comprehensive guidelines for the Norwegian school meal were introduced, to raise more awareness in this regard [13]. Among the Nordic countries, Denmark has similar school meal arrangement as Norway (i.e. packed lunch from home), while Sweden and Finland have served a warm school meal every day for several years, free of cost for the children [14].

Only one intervention study in Norway has previously assessed the effect of serving a free school lunch on dietary habits and body mass index (BMI) among 9th graders [15]. The intervention period was relatively short (4 months). Ask et al. found that serving a free, healthy school lunch did not lead to an improved intake of fruit, vegetables, low-fat milk and whole-grain bread, nor reduced intake of unhealthy snacks, and BMI increased among the boys in the intervention group compared to the control group, but not among the girls [15]. No moderating effects of SES was assessed.

School-based interventions intend to decrease social inequalities among children and adolescents, but sometimes the opposite may occur; children of higher educated parents seem to benefit more from interventions than children of lower educated parents [16]. The aim of this study was to assess the effect of 1 year of serving a free school meal on dietary habits at school and weight status among 10–12-year- old’s in Norway, as well as to investigate the moderating effect of SES on the intervention effects.

## Methods

This present study is part of the School Meal Project in Southern Norway [17] which had a non-randomized design with one intervention group and one control group. The baseline data was collected in August/September 2014, and follow-up data was collected in January 2015 and in June 2015. The school children answered a food frequency questionnaire at school, and height, weight and waist circumference were measured at all time points.

### Content of the intervention

A healthy, cold school meal free of charge was served every school day for 1 year to the children in sixth grade at one elementary school in Southern Norway. The intervention has previously been described by Illokken et al. [17], and was based on the current national dietary guidelines in Norway and consisted of whole-grain bread, healthy spread and fruit and vegetables (FV). Some children drank milk and the others were encouraged to drink water. The food was served on large platters, and the children helped themselves. The children consumed the food together around one or two tables in the classroom, which represented a social arena for the meal. A teacher was always present during the meals.

### Sample and procedure

A local cook prepared and served the healthy school meal every day. In order to make the intervention feasible, a convenience sample was chosen and all children in one school class were allocated either to the intervention or the control group. Two schools were included in the project, and all participating children had an age-range from 10 to 12 years (5th – 7th grade) [17]. In one school there was both an intervention group and a control group, and in the other school three was only a control group. Both schools were located in a rural area in the same county, and they were similar in school size. Active parental consents were required by the Norwegian Centre for Research Data and before baseline measurements, written consents to participate in the School Meal project were collected. The participating children were made aware of the possibility to withdraw from the project. Project workers were present when the children filled out the questionnaires (by pen and paper) during a school lesson, in order to clarify possible misapprehensions. The children were asked to consider their eating habits for the previous 2 weeks when filling out the questionnaires.

### Measures

Diet was assessed by a food frequency questionnaire. The questions had six different response alternatives, ranging from “never” to “every day”, and included questions on food habits at school with the usual packed lunch (baseline and follow-ups in the control group) and the served school meal (follow-ups in the intervention group). Diet was assessed with items derived from previous validated questionnaires [17].

A Healthy food score (HFS) based on 13 selected food items was developed [17]. The food frequency questionnaire included more than the selected 13 items; however, they were chosen in order to differentiate between the children in the sample who had a healthier intake at lunchtime compared to those who had an unhealthier intake. Hence, both healthy and unhealthy food items were included in the score. Healthy food items, e.g. whole-grain bread, fish, berries and FV and unhealthy food items, e.g. white bread, noodles, chocolate spread, crackers and pancakes were included to investigate possible change in the consumption of these. Response alternatives of healthy and unhealthy food items were recoded into healthy (=one) or unhealthy (=zero) depending on frequency of intake (Table 1). Missing values were included as zero. The total food score included a summed value from all the 13 recoded scores. The HFS ranged from one to 13, i.e. a higher HFS resembled a higher intake of healthy food items.

**Table 1:** Healthy and unhealthy categories according to intake of 13 food items^a^

Food item	Score 1 (“healthy”)	Score 0 (“unhealthy”)
	Times per week	Times per week
Wholegrain bread	≥4	< 3
White bread	0	≥1
Crackers	0	≥1
Noodles	0	≥1
Pancakes	0	≥1
Buns, waffles, muffins	0	≥1
Chocolate spread	0	≥1
Fish spread	≥1	0
Jam	0	≥1
Fruits	≥4	< 3
Berries	≥1	0
Vegetables	≥4	< 3
Nuts/almonds	≥1	0

^a^0 = never, 5 = every school day. Wholegrain bread includes wholegrain bread rolls; white bread includes white bread rolls

Parents’ level of education was assessed in the parent questionnaire by two items: “What is your highest level of completed education?” with four response options; “primary school (elementary school or lower secondary school)”, “upper secondary school”, “3-4 years of college or university” and “5 or more years of college or university” and “what is your spouse/partner’s highest level of completed education?”. The response options were the same as the previous item, but also included; “I do not have a spouse/partner”. The parents’ educational level was a proxy for SES. Both scores were combined and dichotomized into “low SES” (both parents having completed primary school and upper secondary school) and “high SES” (at least one parent having completed 3–4 years and more than 5 years of college/university) [18].

Body height, weight, and waist-circumference (WC) of the children were measured at school. The methodology has previously been published [17]. BMI z-scores were calculated according to the International Obesity Task Force criteria (IOTF) [19].

### Statistical analysis

Preliminary analyses consisted of descriptive statistics of sample characteristics and normality of the outcome variables was checked. Participants’ characteristics at baseline were compared by independent sample t-tests for continuous variables and by chi-square tests for categorical variables to detect baseline differences between the control and the intervention group. No drop-out analysis was conducted given that few children were lost to follow-up.

Because baseline characteristics did not differ significantly between intervention and control group apart from gender, which is included as a moderator, they were not used as covariates in further analyses. Intervention effects on children’s Healthy food score, BMI z-scores, and waist circumference were examined by conducting a Repeated Measures Multivariate ANOVA with time as within factor (differences between baseline and follow-up 1 and follow-up 2, and between follow-up 1 and follow-up 2) and condition (intervention group, control group) as between factor. To examine potential moderating effects of children’s gender (boys versus girls) and parental SES (lower versus higher SES), a three-way interaction effect (time*condition*moderator) was investigated for each outcome. The Repeated Measures Multivariate ANOVA was performed using IBM SPSS Statistics 24.0. All analyses used complete cases for the outcome variables (excluding the children that had missing outcome data for one of the follow-ups), and *p*-values of < 0.05 were considered significant.

## Results

### Sample characteristics

**Fig. 1:**
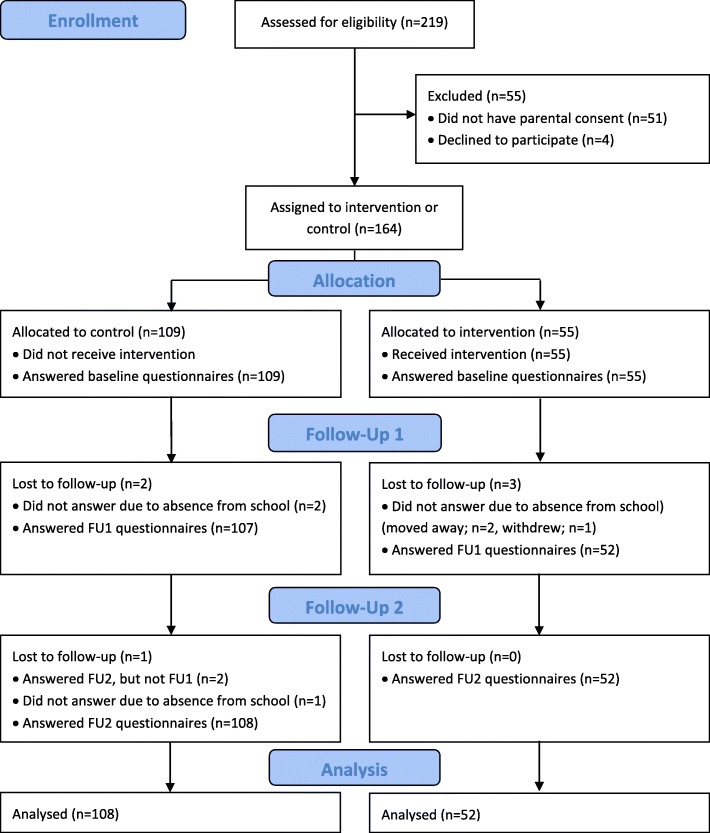
Flow Diagram Children

A total of 219 children were invited to join the project and 168 of the invited children received active parental consent, however four children chose not to participate. The study sample thus consisted of 164 children at baseline (participation rate 75%). The intervention group consisted of 55 children from 6th grade (participation rate 96%), while the control group consisted of 109 children from 5th, 6th and 7th grade (participation rate 67%) at baseline (T0). A total of 154 parents participated at baseline (participation rate 70%). In the first follow-up (T1), 159 children participated (participation rate 73%). In the second follow-up (T2), 160 children participated (participation rate 73%). The reason why a few children were lost to follow-up in T1 and T2 are described in Fig. 1.

Baseline characteristics of the study sample are presented in Table 2. Both groups are comparable at the baseline, apart from significant gender differences. The intervention group included more boys and the control group more girls.

**Table 2:** Comparison of baseline characteristics of intervention and control group

Characteristic	Intervention group (*n* = 52)*	Control Group (*n* = 106)*	Group comparison
*Socio-demographic variables*			
Gender child, n (%)			**χ**^**2**^ **= 4.151**
Boys	33 (63.5)	49 (46.2)	
Girls	19 (36.5)	57 (53.8)	
Age child, mean (SD)	10.60 ± 0.32	10.64 ± 0.92	t = 0.379
SES, n (%)			χ^2^ = 1.201
Lower	24 (46.2)	37 (34.9)	
Higher	27 (51.9)	61 (57.5)	
	1 missing	8 missings	
*Outcome variables*			
Healthy food score, mean (SD)	6.79 ± 2.54	7.24 ± 2.33	t = −1.102
BMI z-score, mean (SD)	0.65 ± 1.13	0.63 ± 0.98	t = −0.136
Waist circumference, mean (SD)	68.56 ± 9.38	67.16 ± 8.45	t = −0.939

*p* < 0.05 indicated in bold

*Only complete cases at all three timepoint were included in the descriptives

### Intervention effects on children’s healthy food score and anthropometrics

The Repeated Measures Multivariate ANOVA analysis showed a significant effect of the intervention on children’s outcomes between baseline and follow-up 2 (*F* = 2.409, *p* < 0.001), and between follow-up 1 and 2 (*F* = 8.209, *p* < 0.001). The Univariate analyses indicated a greater increase in the Healthy food score of the intervention group between baseline and follow-up 1 (*F* = 4.184, *p* = 0.043) and follow-up 2 (*F* = 10.941, *p* = 0.001) compared to the control group. No significant differences were found for the Healthy food scores between Follow-up 1 and 2 of both groups. Unexpectedly, the two-way interaction effects showed that the intervention-children had a significant increase in BMI z-scores between baseline and follow-up 2 (*F* = 10.007, *p* = 0,002) and between follow-up 1 and 2 (*F* = 22.245, *p* < 0.001) compared to a decrease in the control-children. No significant differences were found for the changes in BMI z-scores from baseline to follow-up 1. In addition, no significant intervention effects were found on waist circumference between baseline and both follow-ups. The results of these analyses can be found in Table 3.

**Table 3:** Results of the Repeated Measures (Multivariate) ANOVA analysis

Children’s outcomes from T0 – T1												
Multivariate					Time x Group							
					F	p						
					2.409	0.069						
Univariate		n	T0	T1	Time x Group		Time x Group x Gender		Time x Group x SES			
			Mean (SD)	Mean (SD)	F	p	F	p	F	p		
Healthy food score	IG	52	**6.72 ± 2.52**	**8.28 ± 2.73**	**4.184**	**0.043**	0.049	0.825	1.183	0.279		
	CG	102	**7.28 ± 2.35**	**7.97 ± 2.23**								
BMI z-score (IOTF-based)	IG	52	0.59 ± 1.10	0.55 ± 1.15	1.950	0.165	0.342	0.560	0.072	0.789		
	CG	102	0.64 ± 0.98	0.63 ± 0.96								
Waist circumference	IG	52	68.42 ± 9.42	68.77 ± 9.28	2.618	0.108	3.293	0.072	0.216	0.643		
	CG	102	67.18 ± 8.51	68.34 ± 8.72								
Children’s outcomes from T0 – T2												
Multivariate					Time x Group							
					F	P						
					**8.452**	**< 0.001**						
Univariate		n	T0	T2	Time x Group		Time x Group x Gender		Time x Group x SES			
			Mean (SD)	Mean (SD)	F	p	F	p	F	p		
Healthy food score	IG	49	**6.73 ± 2.54**	**9.00 ± 1.59**	**10.941**	**0.001**	0.105	0.747	**6.228**	**0.014**		
	CG	101	**7.29 ± 2.36**	**8.24 ± 2.14**								
BMI z-score (IOTF-based)	IG	49	**0.60 ± 1.11**	**0.64 ± 1.07**	**10.007**	**0.002**	1.410	0.237	1.465	0.228		
	CG	101	**0.64 ± 0.98**	**0.50 ± 0.96**								
Waist circumference	IG	49	68.44 ± 9.52	70.11 ± 9.16	0.608	0.437	0.096	0.757	0.247	0.620		
	CG	101	67.27 ± 8.51	69.35 ± 8.78								
Children’s outcomes from T1 – T2												
Multivariate					Time x Group	Time x Group						
					F	p						
					**8.209**	**< 0.001**						
Univariate		n	T1	T2	Time x Group		Time x Group x Gender		Time x Group x SES			
			Mean (SD)	Mean (SD)	F	p	F	p	F	p		
Healthy food score	IG	49	8.35 ± 2.71	9.00 ± 1.59	0.848	0.359	0.256	0.614	1.552	0.215		
	CG	101	7.96 ± 2.24	8.24 ± 2.14								
BMI z-score	IG	49	**0.55 ± 1.16**	**0.64 ± 1.07**	**22.245**	**< 0.001**	0.884	0.349	1.505	0.222		
	CG	101	**0.64 ± 0.96**	**0.50 ± 0.96**								
Waist circumference	IG	49	68.81 ± 9.37	70.11 ± 9.16	0.746	0.389	3.077	0.082	0.000	0.998		
	CG	101	68.41 ± 8.74	69.35 ± 8.78								

*IG* intervention group, *CG* control group, *T0* baseline, *T1* follow up1, *T2* = follow up2; *p* < 0.05 indicated in bold

### Moderating effects of children’s gender and parental SES

From baseline to follow-up 2, the time*group*SES interaction effect was significant for the Healthy food score. Stratified analyses showed a significant time*group two-way interaction for the Healthy food scores in the lower SES group (*F* = 7.762, *p* = 0.007) compared to a non-significant two-way interaction effect in the higher SES group. The intervention-children with a lower SES had a significantly higher increase in Healthy food score between baseline and follow-up 2 than the control-children with a lower SES (i.e., a difference of 2.8 compared to 0.94). No significant difference in Healthy food score was found between the intervention and control children with a higher SES. The results of these moderation analyses can be found in Table 3, the stratified analyses are not shown.

## Discussion

In this study we found that serving a free school meal for one year increased children’s intake of healthy foods, especially among children with lower socio-economic status. We found a greater increase in the Healthy food score of the group receiving free school lunch between baseline and follow-up 1 and between baseline and follow-up 2 compared to the control group. This indicates that the children in the intervention group changed their diet in a more favourable way during the intervention period compared to the control group. This contradicts the findings of Ask and colleagues [15] which found that a free, healthy school lunch (wholemeal bread, different kinds of unsweetened spread, low-fat milk and fruit/vegetables) to 9th graders for 4 months did not improve the food score, i.e. intakes of fruit, vegetables, low-fat milk and wholegrain bread, or reduce the intake of snacks, sugar-sweetened beverages and candy/chocolate [15]. Our findings are in line with two other studies examining school meal and eating habits among school children in Finland, which found that intake of free school meals was associated with healthier eating habits, both at school and outside school [20, 21].

No significant differences were found for the Healthy food scores between follow-up 1 and 2 of both groups. This may be due to that fact that the positive changes had already happened in the intervention group from baseline to follow-up 1, and that no differences were expected for the control group. Nudging child diet in the right direction is optimal for child development and health. According to Heckman and colleagues [22, 23] small positive changes early in life are valuable for the individual’s future health and for the society in a public health perspective. A specific cost-benefit for a school meal has not been calculated. However, calculations regarding increasing child intake of one fruit or vegetable per day done by Norwegian health authorities, show that there are large health and economic benefits [24]. Our results are the first to show dietary improvements after a free school meal in Norway among both boys and girls. A previous intervention in Norway where free breakfast was served to 10th graders for four months, reported an improved food pattern among boys only in the intervention group compared to the control group [25]. However, since this was a breakfast intervention compared to lunch, the results are not completely comparable.

The intervention-children with a lower SES had a higher increase in Healthy food score between baseline and follow-up 2 than the control-children with a lower SES. No significant difference in Healthy food score was found between the intervention and control children with a higher SES. This finding may indicate that by introducing a healthy school lunch, social inequalities can be reduced. There are large socioeconomic differences in diet and health, adding to the burden of lower SES families [26–28]. As interventions have been reported to increase social inequalities in health [16] with children of higher educated parents benefitting most from interventions, this study showed the opposite by only having effect in lower socio-economic groups. As the main public health policy goal is to reduce social inequality in health in Norway, this study shows that a free healthy school meal may reduce such differences. Such a program will need change of policy and increased funding to be conducted. As milk and fruits (in some schools) are available to buy at Norwegian schools, one could start by including healthy meals as a part of these structures. However, in general, we know that interventions to promote healthy eating is more effective in lower SES groups if it is free or at reduced price [29]. Our results are in line with those seen for free fruit and vegetables at school, namely an increase in intake and reduction in social inequality [30, 31]. However, the overall quality of diet was improved in our study, not just FV, which would be even more beneficial. Findings from a systematic review in Europe indicate that children from lower SES may profit from school-based interventions promoting a healthy diet [32].

Unexpectedly, the two-way interaction effects showed that the intervention-children had a significant increase in BMI z-scores between baseline and follow-up 2 and between follow-up 1 and 2 compared to a decrease in the control-children. No significant differences were found for the changes in BMI z-scores from baseline to follow-up 1. Ask and colleagues [15] found that BMI for girls in the intervention group (free school lunch for 4 months) did not increase, while a significant increase was seen among the boys in both intervention and control group. Both the Ask study and the present study have few participants, and this result may be due to chance. The current project had no intention of children reducing their weight and at the age of 10–12, children are in growth, so normally we would not expect significant changes in BMI after one year. The purpose was to contribute to healthy eating at school and long-term healthy weight status. In this regard, one year is not long-term. Also, the children were served lunch from a buffet and this may have contributed to larger portions in the beginning. It is difficult to say whether these differences indicate a healthy or unhealthy weight trajectory for the intervention group. The same associations were not seen regarding waist circumference where no change was found. The effect of a free meal on weight should be investigated further.

### Strengths and limitations of the study

The duration of the intervention (serving a free healthy school meal) was one full school year, which may be considered as a long-lasting intervention. The design with an intervention group and a control group, and the high participation rate are other strengths. Trained project workers collected all data in the study to ensure consistency and the items in the questionnaires were validated.

There are also some limitations to our study. The non-randomized study design and the fact that the intervention group was located at the same school as part of the control groups represents a substantial limitation. However, the children in the intervention group were in a totally different part of the school building than the control group, minimizing the chance of confounding, e.g. when the school meal was brought to school every day. Differences in age as well as differences in group size between the intervention group and the control group constitute another limitation. The present study is based on self-reported data relying on memory which could introduce response bias [33]. Also, all the children were aware of the purpose of the study, and this might have influenced their answers. The representativeness and generalizability of this study might have been influenced by these limitations, and the results should therefore be interpreted with caution.

## Conclusion

This study finds that at free healthy school meal for one year improves overall diet at school especially among those needing it the most; children from lower socio-economic status. The study also reports an increase in BMI z-score for the intervention group, however no change in waist circumference. These results should be studied further. Regardless of relation to weight, nudging the diet in the right direction has large potential health benefits for the children and economic benefits for the society. Free school meals may have a great potential for health promotion and on improving future public health measures among children.

## Data Availability

The datasets used and/or analysed during the current study are available from the corresponding author on reasonable request.

## Abbreviations

BMI: Body mass index
CG: Control group
IG: Intervention group
SES: Socio-economic status
T0: Baseline
T1: follow up1
T2: follow up2
